# Great Thermal Conductivity Enhancement of Silicone Composite with Ultra-Long Copper Nanowires

**DOI:** 10.1186/s11671-017-2237-z

**Published:** 2017-07-25

**Authors:** Liye Zhang, Junshan Yin, Wei Yu, Mingzhu Wang, Huaqing Xie

**Affiliations:** 1 College of Engineering, Shanghai Polytechnic University, Shanghai, 201209 China; 2 Shanghai Yueda Advanced Materials Technology Co. Ltd., Shanghai, 201209 China

**Keywords:** Nanowires, Ultra-long, Copper, Thermal conductivity, Silicone composites

## Abstract

In this paper, ultra-long copper nanowires (CuNWs) were successfully synthesized at a large scale by hydrothermal reduction of divalent copper ion using oleylamine and oleic acid as dual ligands. The characteristic of CuNWs is hard and linear, which is clearly different from graphene nanoplatelets (GNPs) and multi-wall carbon nanotubes (MWCNTs). The thermal properties and models of silicone composites with three nanomaterials have been mainly researched. The maximum of thermal conductivity enhancement is up to 215% with only 1.0 vol.% CuNW loading, which is much higher than GNPs and MWCNTs. It is due to the ultra-long CuNWs with a length of more than 100 μm, which facilitates the formation of effective thermal-conductive networks, resulting in great enhancement of thermal conductivity.

## Background

Copper is the third most widely used commercial metal (after iron and aluminum) and has received an intensive attention due to its availability and outstanding properties such as good strength, excellent malleability, and superior electrical and thermal conductivity [1–3]. Nowadays, considering their excellent chemical and physical properties and potential applications in electronic devices, more and more attentions have been paid to nanostructures [4, 5]. Nanowires are a kind of one-dimensional nanostructured materials which have high aspect ratio, novel properties, and potential applications [6, 7]. As is known to all, the physical as well as the chemical properties of nanowires depend not only on their native material properties but also on their morphologies and structures. In recent years, newly studied nanowires and their applications include silicon nanowire and copper nanowires, and so on [8, 9]. Among various nanowires, copper nanowires (CuNWs) are one of the hottest one due to their excellent electrical and thermal conductivity. Meanwhile, except for electrical and thermal conductivity, it has been confirmed that the morphology of CuNWs also plays an important role in the performance of polymer composites with CuNWs as functional fillers [10–14].

A number of fabrication methods for CuNWs have been developed, including template-assisted synthesis [15, 16], chemical vapor deposition [17], vacuum vapor deposition [18], hydrothermal reduction [13, 14] and so on [19, 20]. However, the above methods are hardly applicable in composite materials because of the limitation in mass production and process complexity. In this paper, large-scale synthesis of ultra-long CuNWs has become a reality through hydrothermal reduction of divalent copper ions using oleylamine and oleic acid as the dual ligands. The CuNWs have been usually used for improving the electrical properties of composite materials [3, 10, 12, 13], but the improvement of composites based on CuNWs was seldom reported. In order to investigate the influence of ultra-long CuNWs on thermal conductivity of polymer composites, silicone composites with different fillers were prepared due to good compatibility of silicone base and easy fabrication of silicone composites. Since graphene nanoplatelets (GNPs) and multi-wall carbon nanotubes (MWCNTs) possess large aspect ratio and superior thermal conductivity [21–24], as a comparison, they were also used for preparing silicone composites. Based on experimental data, the analytical models on polymer composites were developed for simultaneously calculating the thermal property with single or hybrid fillers [25, 26].

Here is a simple method to obtain great thermal conductive silicone composites filled with nanomaterials. There are ultra-long copper nanowires, GNPs, and MWCNTs. It mainly focuses on the morphology features and volume fraction of fillers, which is related to the thermal properties and analytical models of composites. The analysis and comparison of thermal conductivity filled with different fillers are carried out in this work.

## Methods

Hydrothermal method is widely used to prepare nanowires. Lots of publications have reported this method [27, 28]. Now, the ultra-long CuNWs were also synthesized by this method according to the research of Li et al. [11] with some modification. Typically, CuCl_2_·2H_2_O and glucose were added to H_2_O under magnetic stirring. Eighty milliliter of oleylamine, 0.8 mL of oleic acid, and 140 mL of ethanol were mixed together. Afterward, these two solutions were put into a beaker and diluted by water, followed by stirring for 12 h at 50 °C. The mixture was transferred into a Teflon-lined stainless steel autoclave. The autoclave was maintained at a temperature of 130 °C for 12 h. The precipitate was sonicated and centrifuged twice in an ethanol solution containing 2.0 wt.% PVP, then vacuum-dried at 50 °C for 6 h.

GNPs were prepared by three steps [29]. Firstly, natural graphite flakes were intercalated by a mixture of concentrated sulfuric and nitric acids (3:1), and then, the intercalated graphite (washed with distilled water and air-dried) was exfoliated by thermal shock on rapid exposure. The exfoliated graphite was dispersed in acetone by high shear mixing for 30 min followed by bath sonication for 24 h. The GNPs were obtained through filtration and drying at 100 °C for 12 h.

The silicone composites with CuNWs were prepared as follows [30]: the CuNWs with different volume fraction were mixed with the silicone base by using a planetary mixer/deaerator (Mazerustar KK-250S, Kurabo, Japan) for 10 min at room temperature. The mixture was further mixed through grinding to obtain silicone composites with different CuNW loading. As a comparison, the silicone composites with different loading of GNPs and MWCNTs (purchased from Chengdu Organic Chemicals Co. Ltd., Chinese Academy of Sciences) were prepared by the same procedure.

The morphologies of different samples were analyzed by a field-emission scanning electron microscope (SEM; S4800, Hitachi, Japan) and a transmission electron microscope (TEM; 2100F, JEOL, Japan). The crystal structure of the samples was characterized by X-ray diffractometer (XRD) (D8 Advance, Bruker, Germany) equipped with a copper target and nickel filter. X-ray wavelength used in the analysis was 0.154 nm of CuKa. The thermal conductivities of the composites were measured by a thermal conductivity analyzer (C-Therm TCi, C-Therm Technologies Ltd., Canada), which is based upon the modified transient plane source principle. The samples were filled into the mold with a thickness of 2 mm. The thermal conductivity of each sample is tested at least five times to obtained average value. The temperature of test system was controlled at 25 °C by constant temperature box (Shanghai Boxun Industry & Commerce Co., Ltd.).

## Results and Discussion

**Fig. 1:**
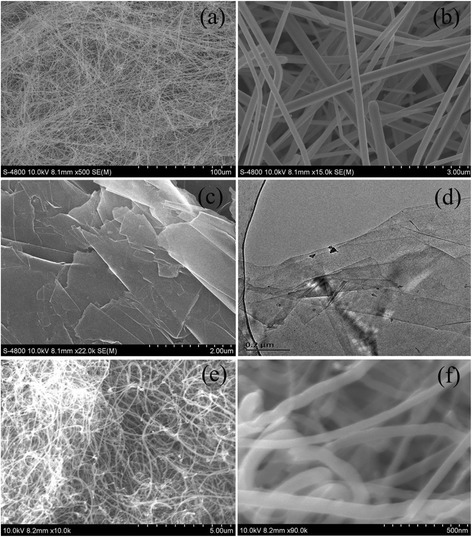
FE-SEM images of different samples of **a** CuNWs, **c** GNPs, and **e** MWCNTs at low magnification and of **b** CuNWs and **f** MWCNTs at high magnification. TEM image of (**d**) GNPs

Figure 1 shows the typical scanning electron microscopy images of three different nanomaterials. The SEM images of ultra-long CuNWs, prepared by hydrothermal method using oleylamine and oleic acid as the dual ligands for 12 h, are displayed in Fig. 1a, b. It is observed that the CuNWs have a main diameter of 250~300 nm, a length of more than 100 μm, and an aspect ratio of 333~400. Besides, the CuNWs have smooth surfaces and are found to be highly flexible as some of them showed bending more than 180° without any fracture. It is clearly revealed that the ultra-long CuNWs are synthesized successfully. In Fig. 1, panels c and d are, respectively, the SEM and TEM images of GNPs. The GNPs show a two-dimensional sheet structure with flat and smooth surfaces and irregular shape. The planar size and thickness of the as-prepared GNPs is in the range of 3–5 μm and ~20 nm, respectively. The typical TEM image of GNPs generally shows wrinkled flakes with edges that are partially folded or scrolled due to the high surface tension needed for the GNPs to maintain its planarity, which shows an aspect ratio of 150~250. As seen from the SEM images of MWCNTs, shown in Fig. 1e, f, their diameter and length are ~50 nm and 10~20 μm, respectively, with an aspect ratio of 200~400. Meanwhile, the MWCNTs exhibit smooth surfaces and good frizzy.

**Fig. 2:**
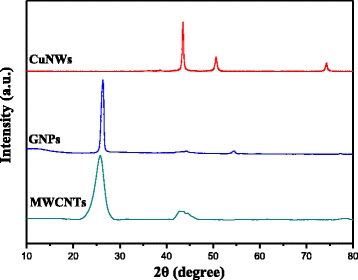
XRD patterns of CuNWs, GNPs, and MWCNTs

The purity and crystal structure of ultra-long CuNWs, GNPs, and MWCNTs were characterized by powder X-ray diffraction, which is shown in Fig. 2. The XRD pattern of CuNWs displays three diffraction peaks, corresponding to the {110}, {200}, and {220} crystal planes of face-centered cubic copper, respectively [11, 14]. Two possible CuO and Cu_2_O impurity phases have not been detected in our ultra-long CuNWs, indicating that the CuNWs is in the form of pure metal. As shown in the XRD patterns of GNPs and MWCNTs, it is clear that the relative intensity and the 2θ of diffraction peaks of GNPs and MWCNTs are similar. Both of them exhibit two characteristic diffraction peaks at 2θ values around 26° and 43° which correspond, respectively, to the {002} and {101} plane diffractions from graphitic carbon [31, 32].

**Fig. 3:**
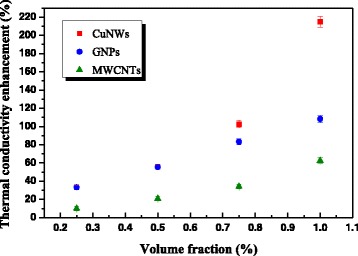
Thermal conductivity enhancements of silicone composites with different fillers as a function of volume fraction

The loading and the intrinsic thermal conductivity of different fillers have significant influences on thermal conductivity and thermal conductivity enhancement of polymer composites. In order to investigate this effect, silicone composites with different fillers were prepared due to good compatibility of silicone base and easy fabrication of silicone composites. Figure 3 is the thermal conductivity enhancement of silicone composites with ultra-long CuNWs, GNPs, and MWCNTs as a function of volume fraction. The thermal conductivity of silicone base is very low, only 0.12 W/mK, while the thermal conductivity of the three composites is greatly improved compared with that of silicone base. The thermal conductivity of the three silicone composites based on different fillers increases with the increase of volume fraction of fillers. The thermal conductivity enhancement of silicone composites with 1.0 vol.% CuNWs, GNPs, and MWCNTs are 215, 108, and 62%, respectively. Quite different from the electrical conductivity of polymer composites, it is a widespread view among polymer composites containing nanomaterials that there is no percolation threshold in thermal conductivity. Yet, there is a turning point to be observed in thermal conductivity of all the three silicone composites, which locates at the loading of 0.5 vol.%. When the loading of filler is lower than 0.5 vol.%, the thermal conductivity of the composites increases slowly with the increase of filler loading, while the thermal conductivity increase significantly faster than before beyond this loading.

**Fig. 4:**
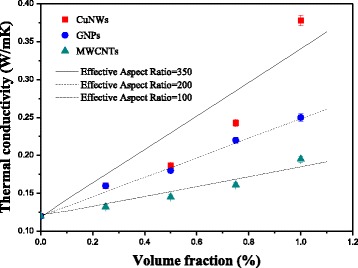
Thermal conductivities of three kinds of fillers in silicone composites with the predictions by the Nielsen model

The thermal conductivity enhancement of silicone composites with 1.0 vol.% CuNWs, GNPs, and MWCNTs are 0.378, 0.251, and 0.195 W/mK, respectively (as shown in Fig. 4). Besides the experimental results, Fig. 4 shows the calculated results obtained by the Nielsen model [33], which consists of the following three equations:

1 $$ \frac{k_c}{k_s}=\frac{1+ AB{\phi}_f}{1-B\varPsi {\phi}_f} $$

2 $$ B=\frac{k_f/{k}_s-1}{k_f/{k}_s+A} $$

3 $$ \varPsi \cong 1+\frac{1-{\phi}_m}{\phi_m^2}{\phi}_f $$

where *k*_*c*_, *k*_*s*_, and *k*_*f*_ are thermal conductivities of the composite, silicone base, and filler, respectively. *ϕ*_*f*_ is the filler volume content, and *ϕ*_*m*_ is the maximum packing fraction of the dispersed fillers. For randomly oriented fillers, *ϕ*_*m*_ equals 0.52 [33]. The parameter is mainly determined by the aspect ratio and orientation of the fillers. According to Table 1 of Ref [33], there is a one-to-one correspondence between the filler aspect ratio *Ar* and the parameter *A*; however, the range of filler aspect ratio is relatively small, only from 2 to 15. In order to calculate the thermal conductivities of the three silicone composites of this work, which contains fillers with large aspect ratios, the following regression equation is obtained by using the five sets of data in Table 1 of Ref [33].

4 $$ A=0.02054+0.5315\times Ar $$

**Table 1:** The physical properties and aspect ratio of three kinds of fillers

Filler	Thermal conductivity (W/mk)	The aspect ratio (SEM and TEM)	Effective aspect ratio (Nielsen model)	Characteristic
CuNWs	398	333~400	350	Hard, linear
GNPs	1000	150~250	200	Planarity
MWCNTs	3000	200~400	100	Smooth, frizzy

For the silicone composites containing CuNWs, the *k*_*s*_ and *k*_*f*_ are set to 0.12 and 398 W/mK, and it is found that the calculation fits well with the experimental results with *A* = 186.1, which corresponds to *Ar* = 350. In the same way, for silicone composites containing GNPs and MWCNTs, the *k*_*f*_ are set to 1000 W/mK [34] and 3000 W/mK [35], and the calculated results fit well with the experimental results with *Ar* = 200 and *Ar* = 100, respectively.

The thermal conductivity of silicone composites containing different fillers depends on the shape, size, and intrinsic thermal conductivity of fillers [30, 36, 37]. It can be seen from Fig. 3 that the thermal conductivity enhancement of silicone composites with CuNWs increases substantially with the increase of the volume fraction than that of silicone composites with GNPs and MWCNTs. The maximum is up to 215% with 1.0 vol.% CuNW loading, much higher than that of silicone nanocomposites with the same GNPs (108%) and MWCNTs (62%) loading. When the volume fraction of fillers is less than 0.5%, the shape, size, and intrinsic thermal conductivity of fillers do not obviously affect the thermal conductivity of silicone composites. This is because the heat-conductive fillers surrounded by silicone base cannot touch each other at low filler loading; hence, the thermal conductivity increases very slowly resulting from high thermal contact resistance inside the composites [30, 36]. While with the loading further increasing, the thermal conductivity of silicone composites with different fillers differs greatly, which indicates that the shape, size, and intrinsic thermal conductivity of fillers have a significant influence on the thermal conductivity improvement of silicone composites. Many studies have reported that the GNPs with superior thermal conductivity and large aspect ratio could greatly improve the thermal conductivity of polymer composites with only a few GNPs [37–39]. And it has stronger ability to enhance the thermal conductivity of polymer composites than MWCNTs [40, 41]. This phenomenon has also been observed in our study. Although the intrinsic thermal conductivity of CuNWs (398 W/mK) is far less than that of GNPs (1000 W/mK) and MWCNTs (3000 W/mK) (as shown in Table 1), the ability of ultra-long CuNWs to enhance the thermal conductivity of silicone composites is stronger than that of GNPs and MWCNTs. It is due to the ultra-long CuNWs with a length of more than 100 μm. The characteristic of CuNWs is hard and linear, which is nothing like MWCNTs (smooth and frizzy). The effective aspect ratio (350) of CuNWs from the Nielsen model is in the range of morphology from SEM and TEM images, which showed the advantage of ultra-long filler on heat transfer. But perhaps because MWCNTs has frizzy and twining structure, the effective aspect ratio (100) from the model is less than that from SEM and TEM. The ultra-long and linear structure facilitates the formation of bridges between themselves and thus to construct some effective thermal conductive networks. These networks provide a low-resistance pathway to heat conduction and increase the overall thermal conductivity of the composite.

## Conclusions

In conclusion, a hydrothermal reduction method of divalent copper ions using oleylamine and oleic acid as dual ligands was used to synthesize ultra-long copper nanowires on a large scale. The CuNWs had a diameter of 250~300 nm, a length of more than 100 μm, and an aspect ratio of 333~400, which was observed by scanning electron microscope. The purity and crystal structure of CuNWs was examined by powder X-ray diffraction. Silicone composites with CuNWs, GNPs, and MWCNTs were prepared to investigate the influence of CuNWs on thermal conductivity of polymer composites. The thermal conductivity enhancement of silicone composite with ultra-long CuNWs increases substantially with the increase of the volume fraction. The maximum is up to 215% with 1.0 vol.% CuNW loading, much higher than that of silicone nanocomposites with the same GNP (108%) and MWCNT (62%) loading. It is due to the ultra-long length and large aspect ratio, which facilitates the formation of effective thermal conductive networks, resulting in great enhancement of thermal conductivity.
